# Drosophila 3′ UTRs Are More Complex than Protein-Coding Sequences

**DOI:** 10.1371/journal.pone.0097336

**Published:** 2014-05-13

**Authors:** Manjula Algama, Christopher Oldmeadow, Edward Tasker, Kerrie Mengersen, Jonathan M. Keith

**Affiliations:** 1 School of Mathematical Sciences, Monash University, Clayton, Victoria, Australia; 2 School of Medicine and Public Health, University of Newcastle, Newcastle, New South Wales, Australia; 3 School of Mathematical Sciences, Queensland University of Technology, Brisbane, Queensland, Australia; Midwestern University, United States of America

## Abstract

The 3′ UTRs of eukaryotic genes participate in a variety of post-transcriptional (and some transcriptional) regulatory interactions. Some of these interactions are well characterised, but an undetermined number remain to be discovered. While some regulatory sequences in 3′ UTRs may be conserved over long evolutionary time scales, others may have only ephemeral functional significance as regulatory profiles respond to changing selective pressures. Here we propose a sensitive segmentation methodology for investigating patterns of composition and conservation in 3′ UTRs based on comparison of closely related species. We describe encodings of pairwise and three-way alignments integrating information about conservation, GC content and transition/transversion ratios and apply the method to three closely related Drosophila species: *D. melanogaster*, *D. simulans* and *D. yakuba*. Incorporating multiple data types greatly increased the number of segment classes identified compared to similar methods based on conservation or GC content alone. We propose that the number of segments and number of types of segment identified by the method can be used as proxies for functional complexity. Our main finding is that the number of segments and segment classes identified in 3′ UTRs is greater than in the same length of protein-coding sequence, suggesting greater functional complexity in 3′ UTRs. There is thus a need for sustained and extensive efforts by bioinformaticians to delineate functional elements in this important genomic fraction. C code, data and results are available upon request.

## Introduction

The fundamental role played by non-protein-coding functional DNA and RNA in cellular processes is no longer contentious. Various lines of evidence have contributed to recognition of its importance. Ever since it became possible to compare two mammalian genomes, it has been clear that far more is conserved than just the protein-coding component [1]. In mammals, unsurprisingly since the encoded proteome is relatively stable, it has been determined that non-coding elements are the predominant source of evolutionary innovation [2], much of which is due to variation in the regulatory architecture [3]. In the human genome, genetic association studies have identified numerous disease-associated genetic variants in non-protein-coding regions [4]–[6]. The ENCODE project, which aims to catalogue all components of the human genome, has found evidence that at least 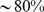 of the human genome is functional, where a functional element is defined as “a discrete genome segment that encodes a defined product (for example, protein or non-coding RNA) or displays a reproducible biochemical signature (for example, protein binding, or a specific chromatin structure)” [7]. Moreover, the ENCODE study identifies that 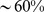 of the genome is included in at least one long (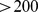 bases) RNA transcript. The ENCODE definition of function, and the 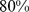 estimate, have been sharply criticised [8], [9] but this debate does not obscure a broad consensus that the functional component of the genome far exceeds the 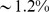 that codes for proteins. It is also becoming increasingly clear that genome-wide transcription is regulated and profoundly complex [10].

The 3′ UTRs of protein-coding genes are a likely source of as yet uncharacterised functional non-protein-coding elements, because this genomic fraction is not only transcribed but also associated with known functional elements (the corresponding genes). There is growing awareness of the crucial importance of 3′ UTRs in post-transcriptional regulation of protein expression (for example [11]). Mutations in 3′ UTRs have been shown to play a crucial role in human health and disease, perhaps as much as that of coding sequences [12]. Our own interest in 3′ UTRs stems from previous work in which we found that a highly conserved component of Drosophila genomes was highly enriched in fragments of sequence from 3′ UTRs [13].

A recent review [14] catalogues a wide range of functional elements in 3′ UTRs. One motif found in 3′ UTRs is the polyadenylation signal with consensus sequence AAUAAA. This signal occurs approximately 10–30 nucleotides upstream of the site at which a pre-mRNA is cleaved prior to polyadenylation, and acts as a protein binding site around which a complex multi-protein assembly forms. A number of other motifs are also known to participate in the process of polyadenylation. More than half of human genes contain alternative polyadenylation sites, resulting in isoforms that differ only in the length of the 3′ UTR. Individual isoforms are also differentially expressed in different cell types and developmental stages. This has important consequences for post-transcriptional regulation, as isoforms with shorter 3′ UTRs tend to be more stable, partly because the shorter isoforms may exclude binding sites for microRNAs. Such binding sites are another common functional element in 3′ UTRs, and in fact most miRNA binding sites are located in 3′ UTRs.

Other key regulatory sequences found in 3′ UTRs include: AU-rich elements and GU-rich elements, to which proteins involved in mRNA degradation bind; a CU-rich element known as the differentiation control element (DICE) to which proteins that inhibit translation initiation bind; other CU-rich elements bound by proteins including polypyrimidine-tract binding protein (PTB), which modulates a variety of mRNA processes including splicing and polyadenylation; CA-rich elements to which proteins that stabilise mRNAs bind; and motifs that form stem-loop structures recognised by specialised regulatory proteins. Repetitive motifs within 3′ UTRs have previously been demonstrated to direct the cellular localisation of mRNA transcripts [15]. Andken *et al.* [15] identify computationally a CAG repeated motif common to many mammalian genes which localise to the dendrites of neurons, and validate experimentally in two specific cases that the correct localisation is dependent on the presence of this motif. Numerous other functional binding sites in 3′ UTRs are known. The database UTRsite maintains a list of experimentally validated functional motifs in UTRs [16].

In this paper, we assess the complexity of 3′ UTRs relative to that of protein-coding sequences, by comparing the extent to which segmental substructures can be detected within these two genomic fractions based on sequence composition and conservation. We argue that the degree of segmental substructure is a useful proxy for functional complexity. We find that segmental sub-structures in 3′ UTRs are shorter on average, more numerous and more varied in type than in protein-coding sequence. Annotation of function in 3′ UTRs will therefore not be complete until it is rather more detailed than the annotation of protein domains in protein-coding sequences. We therefore echo [17] in calling for bioinformaticians to turn their attention to annotation of this important genomic fraction.

Our methodology involves comparing closely related species, which may seem unusual given that functional signatures are more clearly distinguishable from background patterns at greater evolutionary distances. However, we suspect that full elucidation of the functional component of 3′ UTRs may require comparison of closely related species, in addition to conventional comparisons of more distantly related species. Furthermore, it may require consideration of additional data not based on species comparisons, and perhaps unique to individual species. The reason for this is that some functional components of genomes may be ephemeral, that is, may persist in genomes only briefly relative to evolutionary time-scales, perhaps so briefly as to be unique to one extant species.

The existence of such ephemeral functional elements is an inevitable consequence of genetic drift. In finite populations, beneficial mutations are not guaranteed to become fixed, and those that do may subsequently be eliminated in the lottery of genetic drift, particularly if the advantage conferred is slight. Recently evolved functional elements whose integration into the system is not yet optimal are perhaps more vulnerable to random extinction, despite the selective pressures that favour their survival. Such functional turnover is certain to occur in evolving genomes, but the proportion of the human and other genomes currently under ephemeral constraints is not known.

Evidence possibly indicative of ephemeral constraints was uncovered by the ENCODE pilot project [18], which found that not all bases within experimentally defined functional genomic regions show evidence of constraint, and that many functional elements are seemingly unconstrained across mammalian evolution. The authors of that paper proposed that the genome contains a large pool of “neutral elements that are biochemically active but provide no specific benefit to the organism” [18]. We consider that explanation contradictory, since it is intended to address the observation that *functional* elements are seemingly unconstrained, and function implies a benefit to the organism. A more natural conclusion is that a significant proportion of the human genome is subject to ephemeral functional constraints, visible to comparative genomics studies only for closely related species, if at all. More recent ENCODE publications support this latter interpretation, for example finding that elements without detectable mammalian constraint do show evidence of negative selection in primates [7].

Evidence of large-scale turnover of transcription factor binding sites (TFBSs) has been found in Drosophila. Moses *et al.* [19] identified numerous known regulatory binding sites in *D. melanogaster* that were not present in closely related species, including *D. simulans*. There is also mounting evidence that binding of transcription factors (TFs) to seemingly non-functional ‘decoy’ TFBSs has subtle effects on the regulation of target gene expression [20], [21]. Low information content decoy TFBSs are frequently created and destroyed by point mutations and are likely candidates for functional elements under ephemeral constraints. Similarly, post-transcriptional binding sites in 3′ UTRs are mostly low information content sequences that are potentially subject to rapid turnover.

In this paper, we present a sensitive methodology for investigating patterns of conservation and sequence composition in pairwise and three-way alignments of closely related species. Segmentation models are well suited to detecting subtle variations in sequences, and have a long history of use in bioinformatics [22]. In such models, it is assumed that the sequence (usually, but not limited to, DNA) can be partitioned into a series of segments, each with some degree of internal homogeneity. The challenge is to find the positions that delineate the segments (known as change-points). Bayesian models are attractive in these circumstances as they are apt for modelling complex hierarchies, and also provide a natural framework to model uncertainty. The seminal paper for such models is [23], and the approach has recently been developed and extended [13], [24]–[26]. Our Bayesian model and associated Markov chain Monte Carlo (MCMC) sampler were developed for the segmentation of sequences derived from pairwise and multiple alignments.

In earlier work [13], three main classes of conservation level were identified in Drosophila, corresponding to slowly evolving, rapidly evolving and intermediate segments. A more recent analysis involved generalizing the Bayesian segmentation technique to identify patterns of conservation variation in multiple sequence alignments [26]. The method was able to distinguish multiple classes of evolutionary rate; 7 in an alignment of four mammals (including humans) and 9 for an alignment of four drosopholids. The classes were indicative of different degrees of selection acting in a segmented pattern over the genome, the scale of which was much finer than could be attributed to local variations in the neutral mutation rate. These findings indicated a significant problem with the conventionally assumed dichotomy of conservation level (conserved or not) used in many previous analyses based on evolutionary rates [1], [18], [27]–[30]. They also highlighted the importance of sophisticated analyses capable of detecting sub-classes of evolutionary rates, for investigating the vastly complex landscape of evolution. A recent simulation study by the authors [31] demonstrated that this technique does not detect superfluous modes, confirming the above conclusions.

Despite the success of the segmentation approach, it is clear that conservation data alone will not provide sufficient power to detect unique functional signatures. This point is particularly relevant in the analysis of closely related species, where distinctions in conservation level are likely to be fine and difficult to detect. We therefore generalise the segmentation approach for sequences formed from characters of an arbitrary alphabet, making it well suited to incorporate other sequence characteristics that are also suggestive of function. We consider the problem of integrating multiple data types, with the aim of identifying classes on a finer scale than previously. This issue is explored briefly in [13], and raised as area which requires further study. Here we segment and classify the 3′ UTR sequence of *D. melanogaster* based on three data types: conservation relative to one or two other species (based on alignment matches and mismatches), GC content, and transition/transversion rates. We illustrate the methodology for the three pairwise, and one 3-way alignment of *D. melanogaster*, *D. simulans* and *D. yakuba* 3′ UTR sequences. The classes thus identified represent a resource for the future discovery of novel functional elements in Drosophila. We also examined several of our identified classes and investigated the extent to which they display properties consistent with function, and explore potential functional roles of motifs identified to be enriched within the different classes.

## Results, Discussion and Conclusions

We applied our segmentation method to the 3-way alignment and three possible pairwise alignments of 3′ UTRs among the species *D. melanogaster*, *D. simulans* and *D. yakuba*. We also applied the method to four different types of control sequence. To compare the segmentation patterns detected in 3′ UTRs to those of known functional sequences, we segmented a randomly selected portion of the alignment of *D. melanogaster* to *D. simulans* protein-coding sequences, of the same length as the 3′ UTR alignment for that species pair. The requirement that this coding alignment be the same length is necessary because the number of segment classes identified is sensitive to the length of the input sequence. In general, more classes can be detected with a longer input sequence. This process was repeated three times with different coding sequences, to ensure that the results were reproducible. In order to demonstrate the advantage of incorporating multiple data types into an 8-character representation, we segmented a binary representation of conservation (matches/mismatches) in the *D. melanogaster* versus *D. simulans* 3′ UTR alignment. Similarly, we segmented a binary representation of GC content in *D. melanogaster* 3′ UTRs. Lastly, we segmented an artificially generated control sequence with only one class of segments. The artificial sequence was generated using the same overall character frequencies, and to be the same length as the *D. melanogaster* versus *D. simulans* 3′ UTR alignment.

### Model Selection

**Figure 1:**
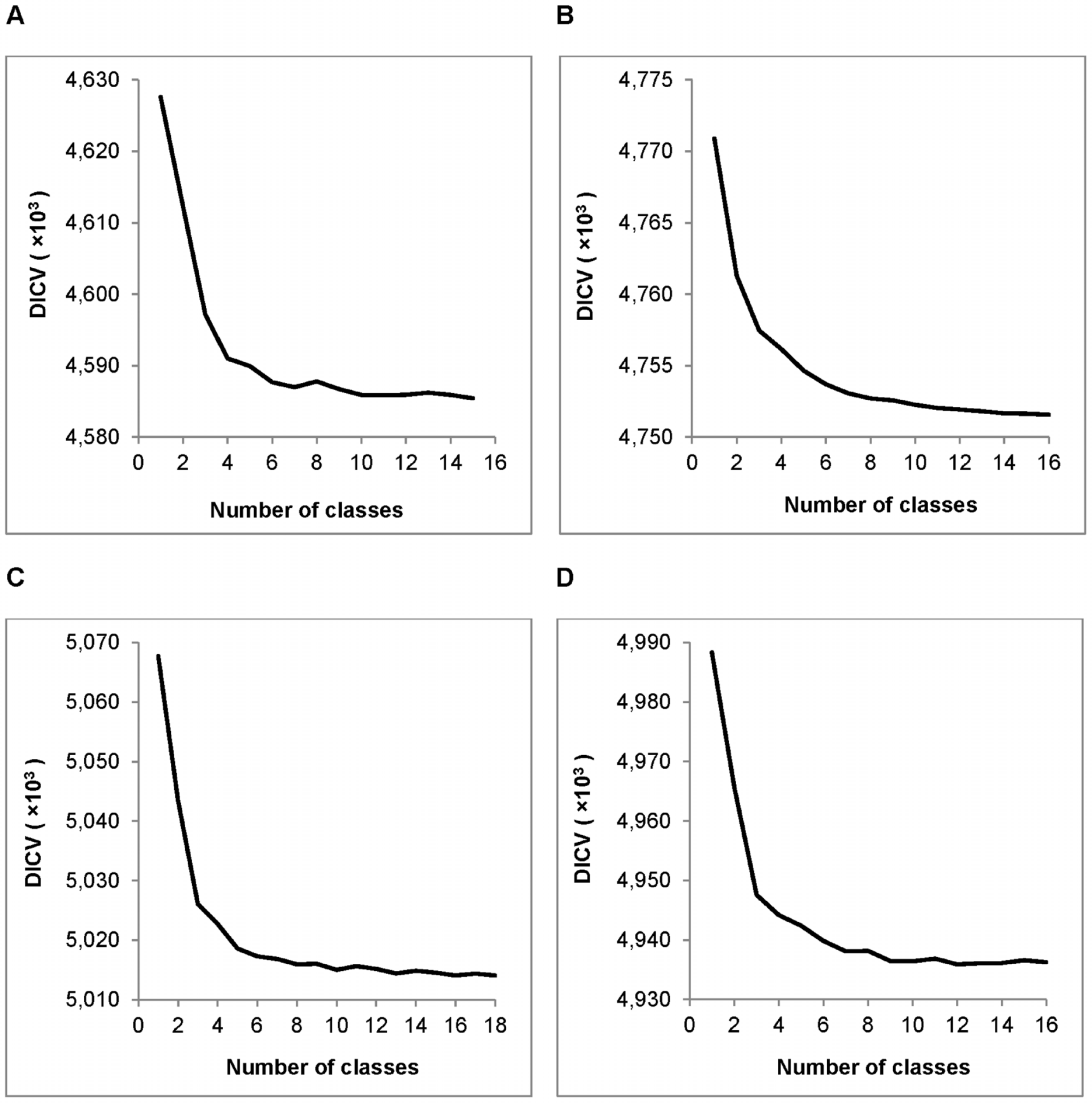
DICV values for segmentation of four alignments. DICV values obtained using a varying number of classes, for four input sequences derived from A) *D. melanogaster* versus *D. simulans* 3′ UTR alignment, B) *D. melanogaster* versus *D. simulans* first coding sequence (Coding 1) alignment, C) *D. melanogaster* versus *D. yakuba* 3′ UTR alignment and D) *D. simulans* versus *D. yakuba* 3′ UTR alignment.

At present our segmentation algorithm requires the user to specify the number of segment classes 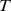. Separate segmentations were therefore performed for each value of 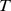 in the range 1–20. Two different procedures were then applied to select the number of classes for each alignment; investigating Deviance Information Criterion V (DICV) values (Procedure 1) and investigating the stability of the classes (Procedure 2). Figure 1 shows plots of the model selection criterion (DICV) versus 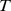 for the segmentations of four 8-character alignment representations. Based on these plots, using Procedure 1, we selected the 12-class model for the *D. melanogaster* and *D. simulans* 3′ UTR alignment (Figure 1A), the 10-class model for the *D. melanogaster* and *D. yakuba* 3′ UTR alignment (Figure 1C), the 12-class model for the *D. simulans* and *D. yakuba* 3′ UTR alignment (Figure 1D), and the 14-class model for the 3-way 3′ UTR alignment (Figure S1).

Using Procedure 2, we selected the 15-class model for the *D. melanogaster* versus *D. simulans* alignment, the 16-class model for the *D. melanogaster* versus *D. yakuba* alignment, the 15-class model for the *D. simulans* versus *D. yakuba* alignment, and the 15-class model for the 3-way alignment. The numbers of classes selected for each sequence by each procedure are summarised in Table 1. In general, Procedure 1 selects a model with fewer classes than Procedure 2.

**Table 1:** Models selected using two procedures.

Alignment	Component	Encoding	Procedure 1	Procedure 2
Dme vs Dsi	UTR	8-char	12	15
Dme vs Dya	UTR	8-char	10	16
Dsi vs Dya	UTR	8-char	12	15
Dme, Dsi, Dya	UTR	32-char	14	15
Dme vs Dsi	Coding 1	8-char	12	12
Dme vs Dsi	Coding 2	8-char	12	12
Dme vs Dsi	Coding 3	8-char	14	14
Dme vs Dsi	UTR	GC alone(binary)	4	2
Dme vs Dsi	UTR	Conservationalone (binary)	2	3

Dme: *D. melanogaster*; Dsi: *D. simulans*; Dya: *D. yakuba*; Procedure 1: Models selected based on DICV values; Procedure 2: Models selected by investigating stability of classes; Coding 1, 2, 3: three different coding sequences.

### Comparison to Control Sequences

Table 1 indicates that twelve to fourteen segment classes with distinct character frequencies can be distinguished in each of the three coding sequence alignments, using Procedure 1 or Procedure 2. The DICV values used for Procedure 1 and one of the three coding sequence alignments are shown in Figure 1B. It is not surprising that such a large number of classes can be detected in coding sequence, given that it consists of numerous sub-units (protein domains) subject to a variety of structural and functional constraints. What is perhaps surprising is that a similar number of classes can be detected in 3′ UTRs, and in fact Procedure 2 consistently identifies a greater number of classes in 3′ UTRs. The implication is that 3′ UTRs contain numerous sub-units subject to an even greater variety of structural and functional constraints than coding sequence. This is in line with the continuing focus in genomics on the significant regulatory and evolutionary role of non-coding sequences, particularly in regard to the regulation of gene expression. Further evidence that 3′ UTRs may have more complex sub-structures than coding sequences is shown in Table 2. The number of change-points estimated in 3′ UTRs is nearly **five times** that estimated for coding sequence, and consequently the average segment length in 3′ UTRs is about one fifth that in coding sequence. Many of these change-points may correspond to the boundaries of functional elements. The values shown in Table 2 were obtained using models selected by Procedure 2, but the same conclusions were reached using models selected by Procedure 1.

**Table 2:** Segmentation characteristics of models selected by Procedure 2.

Alignment	Component	Length	Nfixed	*k*	*L*
Dme vs. Dsi	UTR	2678635	9112	50001	54
Dme vs. Dya	UTR	2486711	8622	53051	47
Dsi vs. Dya	UTR	2481568	8607	51547	48
Dme, Dsi, Dya	UTR	2247759	8260	54523	41
Dme vs. Dsi	Coding 1	2680987	6760	11086	242
Dme vs. Dsi	Coding 2	2681121	6626	10190	263
Dme vs. Dsi	Coding 3	2681284	6463	9982	268

Length: number of alignment columns in the component; Nfixed: number of fixed change-points, corresponding to the boundaries of alignment blocks; *k*: posterior average number of change-points; *L*: posterior average length of segments. Note the length of the coding sequence is equal to that of the 3′ UTRs for the same species pair, once the number of fixed change-points (corresponding to the ends of alignment blocks) is added to the length.

**Figure 2:**
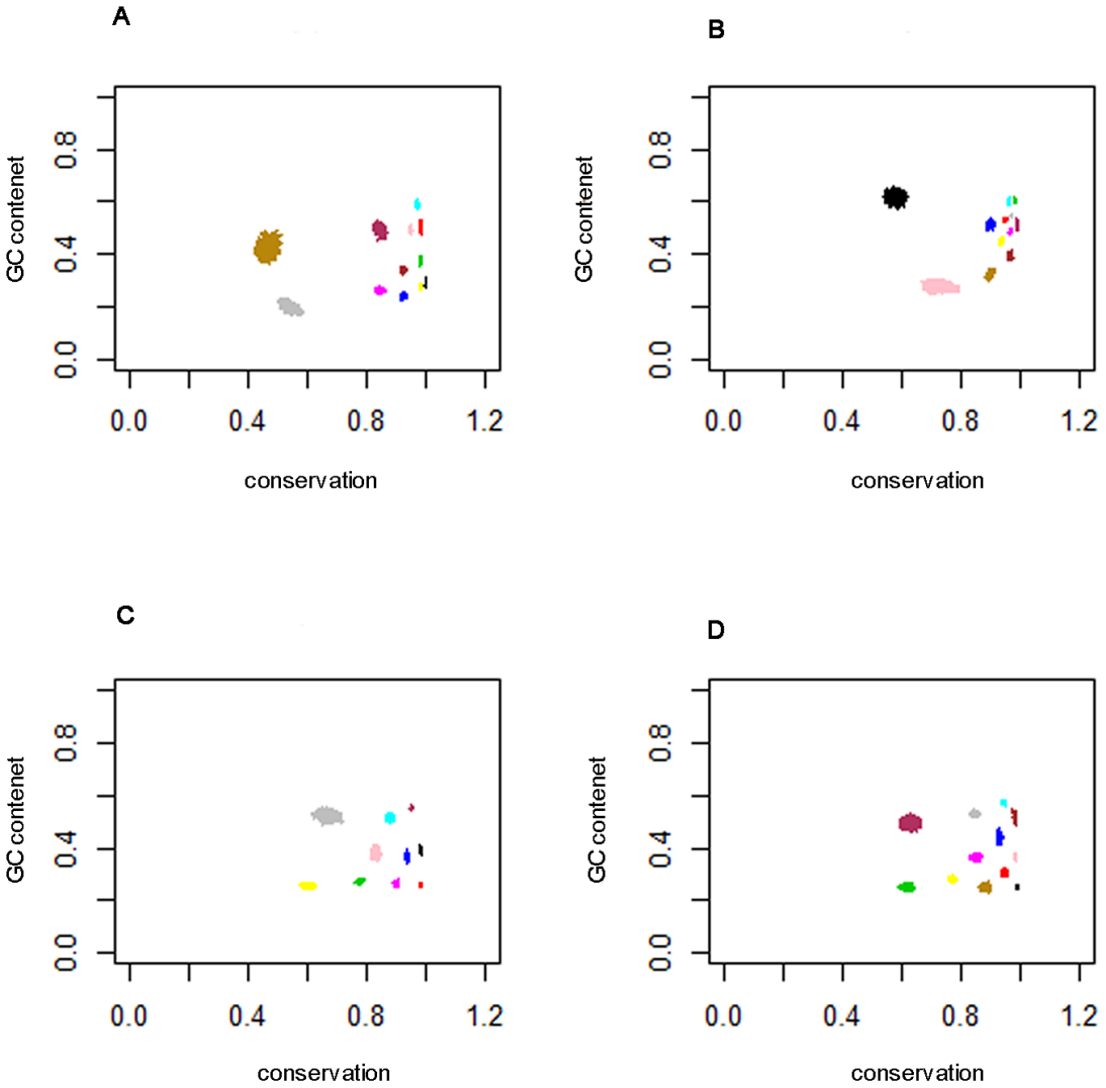
GC content versus conservation level for models selected by Procedure 1. GC content (in the first named species of each pair) versus the proportion of alignment matches, for each model selected by Procedure 1. The different colours represent different classes, and each class is plotted for the post burn-in samples; A) 12-class model for the *D. melanogaster* versus *D. simulans* 3′ UTR alignment, B) 12-class model for the *D. melanogaster* versus *D. simulans* first coding sequence (Coding 1) alignment, C) 10-class model for the *D. melanogaster* versus *D. yakuba* 3′ UTR alignment and D) 12-class model for the *D. simulans* versus *D. yakuba* 3′ UTR alignment. This is a crude diagnostic used to determine if the model has converged in distribution and also indicates how well separated the classes are.

**Figure 3:**
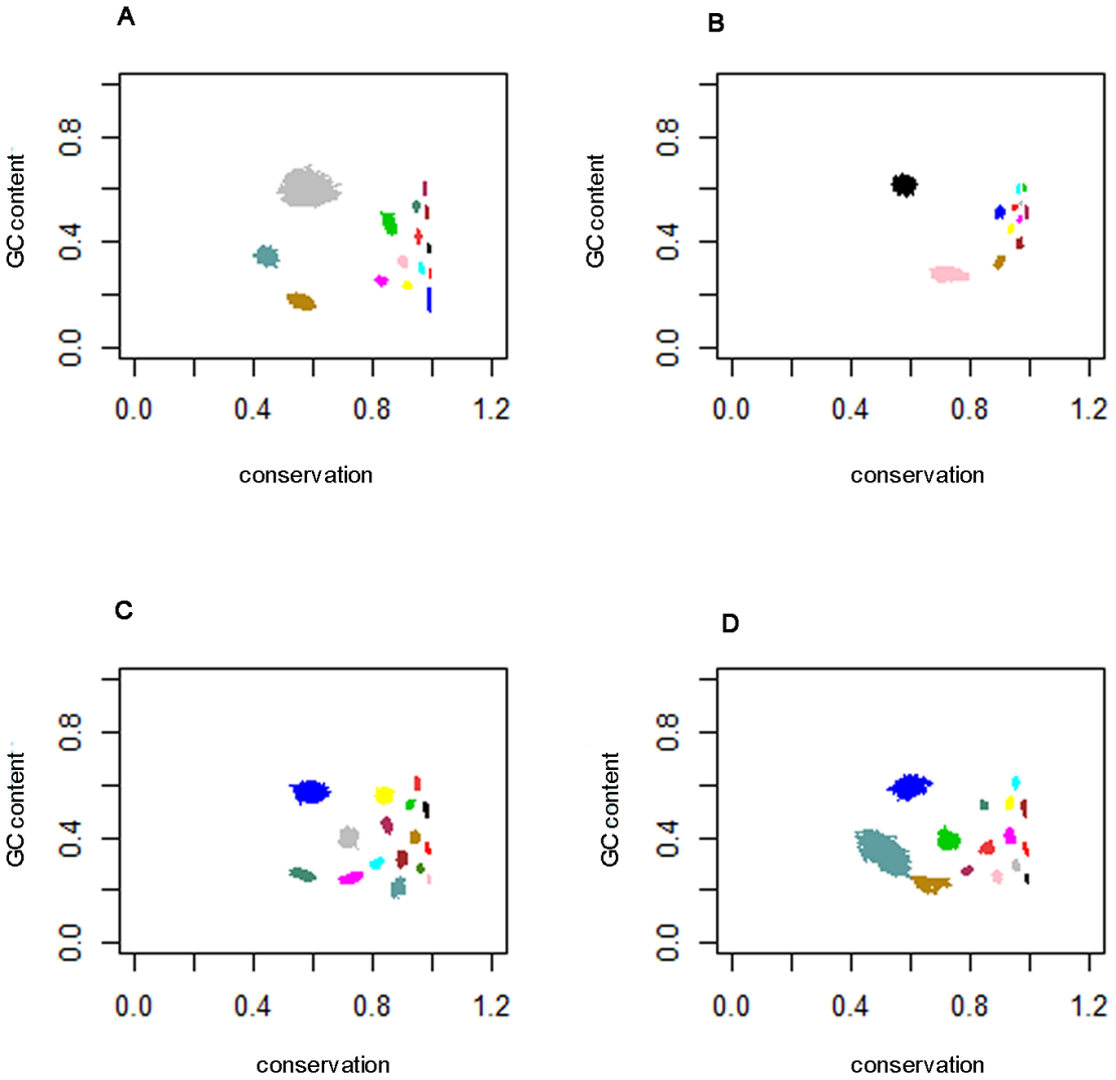
GC content versus conservation level for models selected by Procedure 2. GC content (in the first named species of each pair) versus the proportion of alignment matches, for each model selected by Procedure 2. The different colours represent different classes, and each class is plotted for the post burn-in samples; A) 15-class model for the *D. melanogaster* versus *D. simulans* 3′ UTR alignment, B) 12-class model for the *D. melanogaster* versus *D. simulans* first coding sequence (Coding 1) alignment, C) 16-class model for the *D. melanogaster* versus *D. yakuba* 3′ UTR alignment and D) 15-class model for the *D. simulans* versus *D. yakuba* 3′ UTR alignment.

Both model selection procedures identified a significantly larger number of segment classes than our previous studies using binary sequence representations of pairwise alignments [13], [25]. Figures 2 and 3 demonstrate why this is the case. The figures show, for the two model selection procedures and the four 8-character alignments, the estimated GC content versus conservation level (proportion of matches) for the classes identified. These are time series plots over the MCMC sample, so the size of the ‘blobs’ is an indication of uncertainty. It is clear from these plots that the use of multiple data types has enabled a greater number of classes to be distinguished, because projection onto either of the ‘GC content’ or ‘conservation’ axes would make many of these classes indistinguishable. The same information for the 3-way alignment of 3′ UTRs is shown in Figure S2.

**Figure 4:**
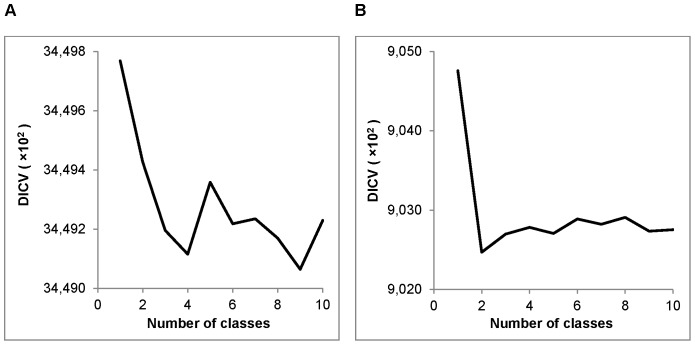
DICV values for segmentation of binary sequences. DICV values versus the number of classes (1–10) for segmentation of: A) the binary representation of GC content in *D. melanogaster* 3′ UTRs, and B) the binary representation of conservation in the *D. melanogaster* versus *D. simulans* 3′ UTR alignment.

To further clarify this point, we compared the number of classes found using the 8-character representation to the number obtained using the binary sequence representing the conservation of *D. melanogaster* relative to *D. simulans* 3′ UTRs (see Table 1). Similarly, we also determined the number of classes found using the binary sequence representing GC content of *D. melanogaster* 3′ UTRs. Figure 4 shows the DICV values with 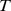 = 1–10 for the segmentation of each of the binary representations. Based on these plots, using Procedure 1, the 4-class model was selected for GC content (Figure 4A), and the 2-class model was selected for conservation (Figure 4B). Using Procedure 2, the 2-class model was selected for GC content, and the 3-class model was selected for conservation. It is clear that the numerous classes identified using the 8-character representation cannot be resolved using either GC content or conservation in isolation.

The final control sequence was artificially generated and was designed to have only one class of segments. Figure S3 shows DICV values for segmentation of this control sequence with 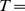 1–5. Note that Procedure 1 correctly selects the 1-class model, thus supporting the use of DICV values for model selection. Figure S4 shows the time-series plot of conservation level versus sample number for segmentations of the artificially generated control sequence with 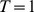 and 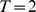. Figure S4A shows the 1-class model is stable, whereas Figure S4B shows that one of the two classes has a widely varying conservation level. This unstable class also had a very low mixture proportion and thus the 1-class model was again selected for the control sequence using Procedure 2. This confirms results of our previous study [31] demonstrating that models selected using DICV do not typically contain superfluous modes, and are generally conservative in the number of components identified.

### Consistency of Segment Classes

In this study, we have used two different model selection procedures to decide how many distinct segment classes can be identified, with Procedure 1 being generally more conservative than Procedure 2, in that it favours fewer classes. The question naturally arises whether the selected number of classes 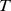 radically alters the classification, or whether the segment classes are consistent in the sense that increasing 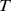 merely results in some classes resolving into two or more subclasses. A similar question arises concerning the consistency of classes identified in the three pairwise alignments and the 3-way alignment. Given that each Drosophila species is involved in two pairwise alignments, one wonders whether comparable classifications result in all three cases.

First, we compared the models chosen by the two model selection procedures, investigating specifically the *D. melanogaster* versus *D. simulans* 3′ UTR alignment. Nine of the classes identified in the 12-class model map directly to individual classes in the 15-class model. The remaining 3 classes from the 12-class model mapped to weighted averages of two classes each from the 15-class model, indicating that the primary difference between the 12-class and 15-class models was the splitting of three classes into two sub-classes each. The results of the mapping are summarised in Table S1: characteristics considered include mixture proportions, conservation levels, GC content and transition/transversion ratio.

Many of the segment classes contain, in the corresponding *D. melanogaster* regions, characteristic tandem repeat sequences detected as highly significant motifs using MEME (see Methods section ‘Class Profiling’), the significance of which are discussed further in the following section. To further investigate the consistency of the 12- and 15-class models, we investigated whether the same characteristic tandem repeats were identified in corresponding classes. In the 12-class model, ten motifs were identified within six classes; within the ten motifs there were six distinct types of motif. In the 15-class model, eleven motifs were identified within eight classes; within the eleven motifs there were six distinct types of motif. Similar motif types to each of the six distinct motif types from the 12-class model were identified in the 15-class model, and in general the motif types found to be common to both models were found in the corresponding classes as identified by the previously mentioned mapping (Table S1). The 15-class model identified two additional motif types not identified in the 12-class model. For this reason, and given that difference between the 12 and 15-class models is only the splitting of three classes, our further analysis of detected motifs focuses on models identified by Procedure 2. A more detailed summary of these results is provided in Tables S2 and S3.

Secondly, we compared the classes identified in the different alignments. Figures 2 and 3 provide an initial indication that the classes detected in the three 2-way alignments of 3′ UTRs are fairly consistent. Figures 3C and 3D in particular, corresponding respectively to alignments of *D. melanogaster* versus *D. yakuba* and *D. simulans* versus *D. yakuba*, are strikingly similar, and many of the classes detected in one alignment can immediately be placed in correspondence with classes detected in the other. Figure 3A, corresponding to the alignment of *D. melanogaster* versus *D. simulans* also shows the same pattern, but corresponding classes appear compressed towards the right of the figure relative to their counterparts in Figures 3C and 3D. This is no doubt due to the shorter evolutionary distance between *D. melanogaster* and *D. simulans*, leading to generally higher conservation levels in most classes. By contrast, the classes shown in Figure 3B, representing the coding sequences alignment, exhibit a pattern distinct from the other three, and it does not appear possible to identify class correspondences.

Further evidence of consistency among the three 2-way 3′ UTR alignments is shown in Table 3. Based on mixture proportions, conservation levels, GC content and transition/transversion ratios, twelve classes were directly comparable among the three 2-way alignments (although the correspondence is more convincing in some cases than in others). There were four cases in which classes were comparable in only two of three alignments, and there were only two cases in which a class was unable to be matched with a class from another alignment. The correspondence between classes identified for different alignments is even more clear when individual character frequencies are compared (Table S5). We also compared the significant motifs detected in the *D. melanogaster* versus *D. simulans* classes (Table S3) to those detected in the *D. melanogaster* versus *D. yakuba* alignment (Table S4). In most cases, classes that correspond in Table 3 were found to contain the same or similar characteristic tandem repeat sequences (Table S5).

**Table 3:** Model comparisons.

Alignment	Class	MP	Conservation	GC content	T/T
Dme vs Dsi	0	15.9%	99%	38%	1.18
Dme vs Dya	1	11.8%	98%	36%	0.96
Dsi vs Dya	1	13.5%	98%	37%	0.97
Dme vs Dsi	1	14.3%	99%	28%	0.80
Dme vs Dya	15	13.8%	96%	28%	0.82
Dsi vs Dya	7	13.6%	95%	29%	0.88
Dme vs Dsi	2	2.0%	86%	47%	0.94
Dme vs Dyaa	7	2.0%	72%	40%	1.03
Dsi vs Dya	2	2.3%	72%	39%	1.00
Dme vs Dsi	3	2.3%	99%	18%	0.50
Dme vs Dya	8	8.5%	99%	24%	0.95
Dsi vs Dya	0	11.0%	99%	25%	0.81
Dme vs Dsi	4	17.1%	96%	30%	0.91
Dme vs Dya	4	7.5%	81%	30%	0.88
Dme vs Dsi	5	2.9%	83%	25%	0.73
Dme vs Dya	13	1.6%	58%	26%	0.71
Dsi vs Dya	11	1.6%	65%	24%	0.71
Dme vs Dsi	6	7.7%	92%	24%	0.67
Dme vs Dya	14	3.9%	89%	22%	0.67
Dsi vs Dya	8	6.9%	89%	25%	0.73
Dme vs Dsi	7	0.3%	58%	60%	0.91
Dme vs Dya	3	0.8%	60%	57%	0.78
Dsi vs Dya	3	0.7%	60%	59%	0.87
Dme vs Dsi	8	8.0%	90%	33%	0.98
Dme vs Dya	10	11.1%	90%	32%	0.92
Dsi vs Dya	12	9.5%	86%	36%	1.03
Dme vs Dsi	9	3.0%	97%	60%	1.48
Dme vs Dya	12	2.3%	95%	60%	1.30
Dsi vs Dya	4	2.2%	95%	61%	1.24
Dme vs Dsi	10	8.2%	98%	51%	1.45
Dme vs Dya	0	4.1%	98%	51%	1.34
Dsi vs Dya	10	4.3%	98%	52%	1.24
Dme vs Dsi	12	11.0%	95%	42%	1.07
Dme vs Dya	11	11.7%	94%	40%	1.11
Dsi vs Dya	5	12.8%	93%	41%	1.08
Dme vs Dsi	13	5.9%	95%	54%	1.32
Dme vs Dya	2	7.9%	93%	53%	1.33
Dsi vs Dya	6	7.8%	93%	53%	1.35
Dme vs Dsi	14	0.7%	44%	34%	0.70
Dsi vs Dya	14	0.5%	52%	34%	0.83
Dme vs Dya	5	3.2%	74%	25%	0.75
Dsi vs Dya	9	6.4%	78%	27%	0.81
Dme vs Dya	6	2.5%	84%	56%	0.95
Dsi vs Dya	13	6.8%	85%	52%	1.06

Comparison of the three models selected by Procedure 2, for each pairwise alignment of 3′ UTRs. MP: mixture proportions; T/T: Transition/Transversion ratio. Class 11 of Dme vs Dsi (MP: 0.7%, Conservation: 56%, GC content: 17% and T/T: 0.5) and the class 9 of Dme vs Dya (MP: 7.5%, Conservation: 85%, GC content: 45% and T/T: 1.1) alignments did not match with other models.

The pattern shown in the plot of GC content versus conservation for the 3-way alignment (Figure S1), upon visual inspection, does not display an obvious similarity to the 2-way alignment plots. However, all but two of the classes can be mapped to classes from the 2-way alignments by considering the frequency of the individual characters within the segment classes (Table S6). While the encodings used for 2-way and 3-way alignments are different, a conserved A or T is represented by the character ‘a’ in both encodings, and a conserved G or C is represented respectively by the characters ‘f’ and ‘v’ in the 2-way and 3-way alignments; thus these characters were used in the comparison of the classes between 2-way and 3-way alignments.

### Exploring Class Content

That such a large number of clearly distinguishable segments and segment classes can be identified in the 3′ UTRs of Drosophila genes is indicative of a surprisingly intricate compositional and mutational complexity. We hypothesize that this complexity results from a wide variety of structural and functional constraints, and we speculate about some of these constraints in this section. We focus on classes from the 15-class model of the *D. melanogaster* versus *D. simulans* 3′ UTR alignment that contain characteristic tandem repeat sequences identified by MEME as highly significant, and which are enriched in elements from the UTRdb, and PicTar annotation databases (see Methods section ‘Class profiling’).

One important concern regarding repetitive motifs is to ensure that they are not in some way artifacts of sequence composition. To test this, we artificially generated 100 control classes for each class from the 15-class segmentation of the *D. melanogaster* versus *D. simulans* alignment which had significant motifs detected (Classes 0, 1, 3, 7, 9, 10, 12, 13; 800 in total). Each control class was generated independently such that the number and lengths of the segments corresponded exactly with one of the observed classes, and such that the frequency of bases was the same as observed in that corresponding class. Each of the control classes was run through MEME. No significant motifs were detected in any of these 800 control classes.

**Figure 5:**
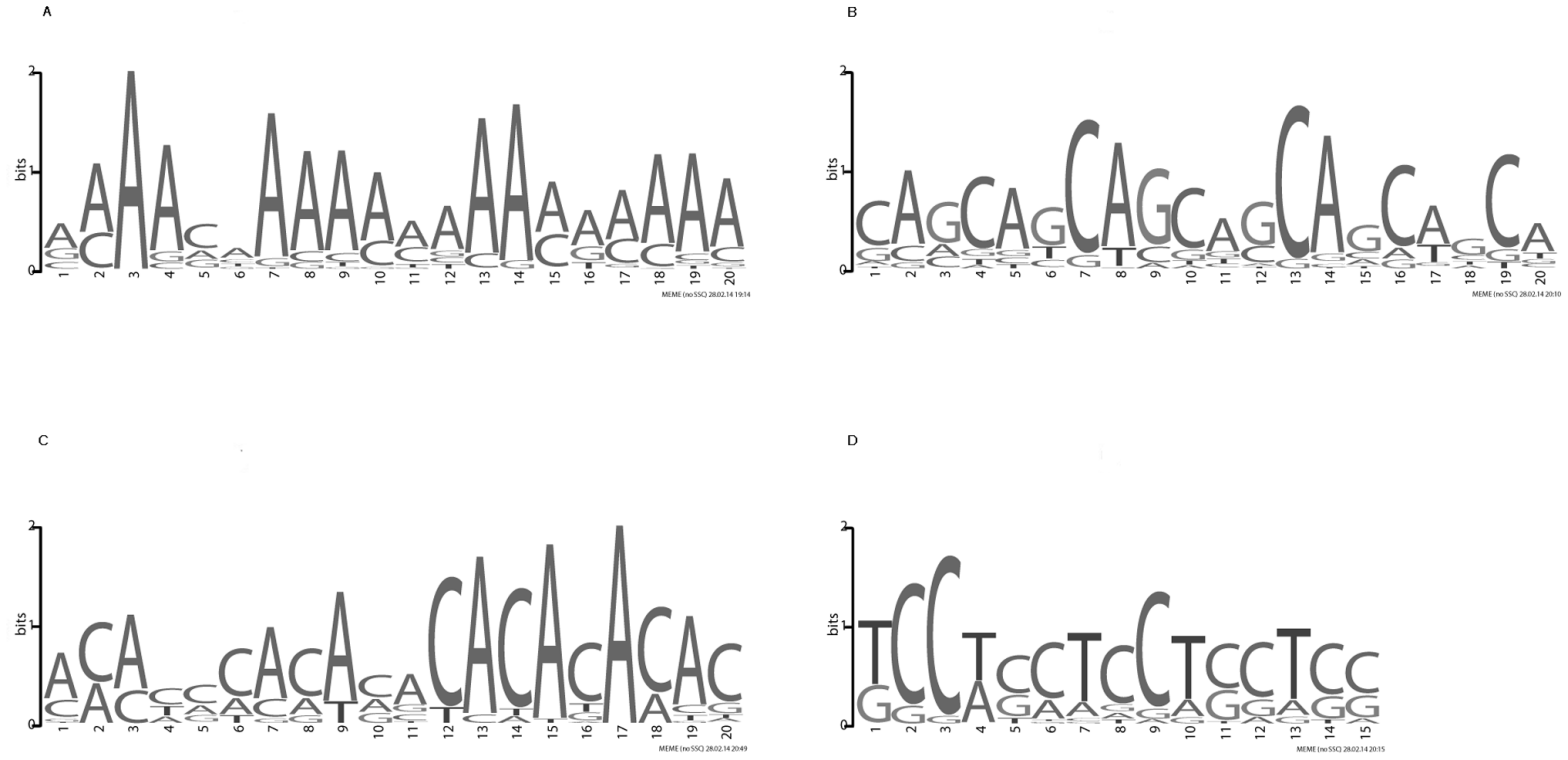
Motifs identified by MEME. Sequence LOGOs for four of the motifs identified by MEME in the 15-class model for the *D. melanogaster* versus *D. simulans* 3′ UTR alignment: A) a polyA motif identified in Class 1, B) a CAG repeat motif identified in Class 9, C) a CA repeat motif identified in Class 12, D) a TCC repeat motif identified in Class 9.

Class 1 had the equal highest proportion of conserved bases (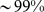) and a relatively low GC content (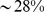). MEME identified two motifs within Class 1 segments: an AT repeat motif common to 171 of 1491 Class 1 segments (E-value: 4.00E-36), and a polyA motif common to 136 segments (E-value: 3.70E-43, Figure 5A). The polyA motif consensus sequence matched the Polyadenylation Signal (PAS, UTRsite motif: U0043), according to the software UTRscan: a program for identifying known UTR regulatory motifs within a given sequence [16]. Class 1 segments were also found to be enriched in the PAS annotation in the UTRdb database (observed: 866, expected: 360, associated p-value: negligible). Given that poladenylation of the 3′ end of mRNAs is near ubiquitous in eukaryotes, it is perhaps unsurprising that our segmentation of 3′UTRs, based on sequence composition and conservation, identified a class of segments enriched in PASs. Cytoplasmic polyadenylation can occur for mRNAs which have been tranlastionally repressed, for example maternally inherited mRNAs which are activated on fertilization [14]. Class 6 segments were found to be enriched in the Cytoplasmic Polyadenylation Element (CPE, UTRsite motif: U0006; observed: 9, expected: 4, associated p-value: 3.26E-9). The median length of 3′ UTRs which contained Class 6 segments was 262 bases (IQR = 480), the shortest of all 15 segment classes, this is perhaps indicative of the inverse relationship between 3′ UTR length and mRNA stability, given that mRNAs requiring cytoplasmic polyadenylation are also required to be stable [14].

Along with Class 1, Class 0 also had the equal highest proportion of conserved bases (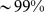), differing on GC content (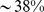). A CAA tri-nucletide repeat motif was identified in Class 0 segments (E-value: 3.0E-34). Both Class 0 and 1 were found to be enriched in multiple miRNA targets, as predicted by PicTar [32]. miRNA targets represent a class of elements found in 3′ UTRs which are important in gene regulation, miRNAs (in cooperation with a protein complex) bind 6–8 mer sites in mRNAs promoting the degradation of the bound mRNA [33] PicTar predictions are partly based on sequence conservation so it is somewhat unsurprising that there is significant overlap between our highly conserved segments classes and PicTar predictions.

Class 9 had the equal highest GC content of the classes (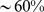), a relatively high proportion of conserved bases (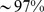), the longest segments (median = 142 bases, IQR = 138), the highest transition/transversion ratio (1.48) and a bias towards the coding end of 3′ UTRs, with a median distance to the coding sequence of 21.5 bases (IQR = 240). Class 10 was notable for a relatively high GC content (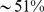), relatively high conservation (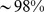), and a relatively high transition/transversion ratio (1.45). Relatively high GC content, high conservation and positional bias are all independently indicative of enrichment in functional elements. MEME identified a CAG tri-nucleotide repeat motif (Figure 5B) in both segment classes, common to 124 of the 298 Class 9 segments and 114 of the 1023 Class 10 segments (E-values, respectively: 5.30E-138, 1.60E-21). TOMTOM identified matches in both the “All vertebrates” and the “All Drosophila” database for both motifs. In the “All Drosophila” database, both CAG repeat motifs matched the binding site of odd, a Drosophila zinc-finger protein. The CAG-repeat motif resembles a repeated E-box: a basic helix-loop-helix (bHLH) binding site with consensus sequence (CANNTG). The matches in the “All Vertebrates” database were both to proteins with bHLH DNA-bonding domains; the Class 9 motif matched the mouse Ascl2 primary binding site (E-value: 2.17E-5), and the Class 10 motif matched the mouse Tcf12 binding site (E-value: 2.47E-5). bHLH protein structures are common to DNA binding proteins involved in transcriptional regulation in all eukaryotes [34]. In Drosophila, twist, acheate-scute, D-mef2 and daughterless are examples of bHLH proteins with well documented regulatory roles that bind E-Box like regulatory elements in order to regulate target gene expression [35], [36]. Furthermore, there are at least 56 known genes in Drosophila coding for proteins with the bHLH DNA binding domain [37].

A CA di-nucletide repeat motif was identified in Class 12, common to 35 of 849 segments (E-value: 3.80E-12, Figure 5C). A possible function for such sites is the documented CA-rich elements (CAREs) which are known to interact with heterogenous nuclear ribonucleoprotein L in order to stabilise mRNAs [14]. In addition, TOMTOM identified matches to three Drosophila zinc-finger protein binding sites in the “All Drosophila” database: klumpfuss, stripe and fruitless (E-values, respectively: 9.43E-3, 2.70E-2, 3.02E-2). TOMTOM also identified matches in the “All Vertebrates” database to the human zinc-finger protein RREB1 and the mouse zinc-finger protein EGR2 binding sites (E-values, respectively: 1.31E-2, 2.55E-2). While many of motifs identified by MEME have similarities with TFBSs, we note that regulatory elements in 3′ UTRs are primarily thought to operate post-transcriptionally and hence to interact with proteins (and miRNAs) that bind RNA, not DNA. The CA-dinucleotide repeat motif was one of two motifs from the 15 class segmentation of the *D. melanogaster* versus *D. simulans* 3′UTR alignment in which TOMTOM identified matches in the “RNA-binding motifs” database. (Recognising a deficiency in knowledge of RNA-binding motifs, the “RNA-binding motif” database was generated by a large-scale experiment for determining binding motifs of known RNA-binding proteins [38]. Synthetic RNA molecules were generated for all possible sequences of length 7, 8 and 9 nucleotides, binding affinity to each motif was measured for 193 unique RNA-binding proteins - 141 with no previously known motif - including 61 from Drosophila.) RNA-binding proteins are known to play a crucial role in gene expression, including roles in splicing, polyadenylation and controlling mRNA stability. One of the most well characterised RNA-binding proteins is the Drosophila Sxl, well known for its role in the complex Drosophila sex determination mechanism [39]. Classes 4, 5, and 6 were enriched in the Sxl binding motif (Table S8). The CA repeat motif matched eleven different RNA-binding motifs in the database, five of which were for Drosophila proteins. Thus it has been shown there are Drosophila proteins which will bind the sequences generating the CA repeat motif. The second motif with a match in the “RNA-binding motif” database is a TCC tri-nucleotide repeat motif, common to 96 of 298 Class 9 segments (E-value: 3.70E-5, Figure 5D). The TCC repeat motif matched the binding site of the human RNA-binding protein SRSF1, a splicing factor.

The positions of segments from each segment class for the segmentation models chosen by Procedure 2 are available in BED format as part of supplementary materials (File S1, S2, S3). A full summary of the motifs identified can be found in Tables S2, S3 and S4, and a full summary of the enrichment of PicTar and UTRdb annotations can be found in Tables S7 and S8. As discussed, several of these repetitive motifs resemble binding sites of common regulatory proteins. While it is possible that TFBSs located within 3′ UTRs could act as enhancer elements [40], in general 3′ UTRs are not considered to play a significant role in transcription activation. It is more likely that these motifs participate in post-transcriptional regulatory interactions with RNA-binding proteins and miRNAs. However, we note in passing that many zinc-finger proteins are capable of binding RNA in addition to DNA, and transcription factors that bind both DNA and mRNAs are known (for example [41]).

### Conclusions

A pairwise alignment can be encoded as an 8-character sequence containing information about sequence conservation, GC content and transition/transversion ratios. A similar approach can be used to encode a three-way alignment as a 32-character sequence. Such sequences can then be segmented and the segments classified according to character frequencies. Here and elsewhere [31] we have shown that DICV provides a method for selecting the number of classes that is conservative in the sense that it does not generally favour models with superfluous classes. We have also proposed a second, less conservative, model selection procedure. Using these encodings, it is possible to distinguish segment classes that could not be resolved on the basis of sequence similarity or GC content considered in isolation. We have therefore proposed the method as suitable for analysing pairwise alignments of closely related species.

An unexpectedly large number of clearly distinguishable segment classes were identified in pairwise and three-way alignments of 3′ UTRs for the species *D. melanogaster*, *D. simulans*, and *D. yakuba*. The number of classes found is comparable to and possibly exceeds the number identified in equal length alignments of protein-coding sequences. The estimated number of change-points in 3′ UTRs exceeds the corresponding estimate for protein-coding sequences by a factor of five. This is suggestive of intricate functional complexity in Drosophila 3′ UTRs, far exceeding that of protein-coding sequences. Similar classes were identified in all three pairwise alignments, suggesting similar constraints are maintained in all three species.

Several of the segment classes we identified were highly enriched in low information content sequences. Although care must be taken to ensure that such motifs are not artifactual, we have used rigorous controls to demonstrate that is not the case here. Moreover, many of the known regulatory sequences in 3′ UTRs have precisely this low information character. We speculate that such regulatory sequences may be frequently created and destroyed in 3′ UTRs, resulting in rapid turnover of functional elements, individual variation in regulatory profiles, and ephemeral conservation. We further speculate that some extended low information content regions of 3′ UTRs may be functional only in the sense that they regularly produce and lose binding sites, thus facilitating changes in regulatory profiles in response to changing selective pressures. A full elucidation of functional elements in 3′ UTRs may therefore require comparisons of closely related species, as well as examination of non-comparative indicators of function.

## Materials and Methods

### Data Transformation

A three-way multiple sequence alignment (MSA) of *D. melanogaster*, *D. simulans* and *D. yakuba* genes was obtained from http://genomics.princeton.edu/AndolfattoLab/w501_genome.html (see also (Hu *et al.* 2013)). The data is made available by the Andolfatto Lab, and incorporates a second generation assembly of the *D. simulans* genome performed in 2012. Annotations of the *D. melanogaster* genome are also provided, and were used to separate the alignments into genic sections, in particular coding regions and 3′ UTRs. The three-way MSA was analysed as three pairwise sequence alignments of *D. melanogaster* to *D. simulans*, *D. melanogaster* to *D. yakuba*, and *D. simulans* to *D. yakuba*.

We used an 8-character sequence representation (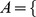
a,b,c,d,e,f,g,h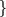) of the pairwise alignments, in which each character in the sequence corresponds to a non-directional mono-nucleotide alignment combination:

Species 1: ATATATATCGCGCGCG

Species 2: ATCGGCTAATCGGCTA

Symbol: aabbccddeeffgghh.

Insertions and deletions relative to *D. melanogaster* are excluded from the representation of the alignment.

For each of the three pairwise alignments, the 8-character sequences for the 3′ UTRs of each gene on chromosome arms 2R, 2L, 3R, 3L were concatenated into a single sequence. Each 3′ UTR segment was separated from the next by a 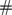 symbol. The *D. melanogaster* versus *D. simulans* alignment of protein-coding sequences was constructed in a similar manner, with each exon separated by a 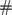 symbol. Three randomly selected subsequences were then selected, each the same length as the *D. melanogaster* versus *D. simulans* 3′ UTR sequence. This was done by choosing a uniform random starting position and then an end position such that that the lengths were the same.

The 3-way alignment of *D. melanogaster*, *D. simulans* and *D. yakuba* was converted to a 32-character sequence representation (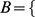
a,b,c,d,e,f,g,h,i,j,k,l,m,n,o,p,q,r,s,t,u,v,w,x,y,z,U,V,W,X,Y,Z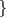).

Species 1: AAAAAAAAAAAAAAAACCCCCCCCCCCCCCCC

Species 2: AAAACCCCGGGGTTTTAAAACCCCGGGGTTTT

Species 3: ACGTACGTACGTACGTACGTACGTACGTACGT

Symbol: abcdefghijklmnopqrstuvwxyzUVWXYZ.

The alignment columns with complementary bases were encoded using the same characters. For example:

Species 1 ‘A’, Species 2 ‘A’, Species 3 ‘A’ =  Species 1 ‘T’, Species 2 ‘T’, Species 3 ‘T’ = ’a’

Species 1 ‘A’, Species 2 ’A’, Species 3 ’C’ =  Species 1 ’T’, Species 2 ’T’, Species 3 ‘G’ = ‘b’.

Two binary sequence representations were also constructed: a binary representation of the GC content in *D. melanogaster* 3′ UTRs (1 for ‘G’ or ‘C’, and 0 for ‘A’ or ‘T’) and a binary representation of conservation in the *D. melanogaster* versus *D. simulans* 3′ UTR alignment (1 for a match, 0 for a mismatch). Both binary sequences involved concatenation in a similar manner as for the 8-character sequences. Note that the binary representations can be recovered from the 8-char representation of the *D. melanogaster* versus *D. simulans* 3′ UTR alignment (as discussed under the heading ‘Assessing Convergence’ below).

### Change-point Modeling

We constructed a Bayesian multiple change point model for the sequences described above. The model is described in detail for binary sequences in previous papers [13], [24], [25] and for larger alphabets in [26], [31]. In summary, this approach estimates positions in the sequence that delineate homogenous segments (known as change-points), the number of which is unknown. The 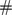 symbol is considered as a fixed change-point. Each segment is drawn from a multinomial distribution with parameters drawn from one of 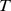 Dirichlet distributions with uniformly sampled probabilities. As the number of classes 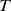 is not known *a priori*, independent runs with values of 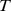 from 1 to 20 were performed. We used an efficient varying-dimensional MCMC technique for simulating from the posterior distribution for the number of change-points, 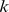, and segment parameters for different numbers of classes. Each model was run for 20,000 iterations and then tested for convergence.

To test our model selection procedures, we also constructed an 8-character control sequence. The sequence was generated such that it was the same length as the *D. melanogaster* versus *D. simulans* 3′ UTR alignment, with fixed change-points in the same positions. Each segment had parameter 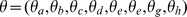 drawn from the same Dirichlet distribution (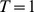), based on the character frequencies of the *D. melanogaster* versus *D. simulans* 3′ UTR alignment.

### Assessing Convergence

To assess convergence of the MCMC sampler in 8-character sequence representation, the mean proportion of no mutations (alignment matches: represented by input symbols ‘a’ and ‘f’) was calculated for each iteration of the sampler:
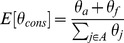



This was plotted against the GC proportions (represented by characters ‘e’, ‘f’, ‘g’ and ‘h’), again calculated for each iteration of the sampler:
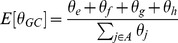



Such plots show a striking trend during the ‘burn-in’ phase of MCMC, at the end of which is a dense ‘blob’ indicating that convergence has occurred. Figures 2 and 3 are examples of such plots, but show only the post-burn-in phase.

For 32-character representation, similar information is given by symbols ‘a’ and ‘v’ for alignment matches and by symbols ‘q’, ‘r’, ‘s’, ‘t’, ‘u’, ‘v’, ‘w’, ‘x’, ‘y’, ‘z’, ‘U’, ‘V’, ‘W’, ‘X’, ‘Y’ and ‘Z’ for GC proportion in species 1 (Figure S2).

### Model Selection

Our current segmentation model assumes that the number of classes (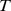) is known; in reality this is not the case. We used two procedures to select the number of classes, after fitting the model for a range of 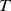. In both procedures, a model containing classes considered to be empty (low mixture proportions) was considered to be an over-fitted model and thus a model with a fewer number of classes would be selected in which the main criterion was still fulfilled (see [42] for a discussion of this approach to model selection).

#### Procedure 1: Investigating DICV

Deviance Information Criterion (DIC) is a criterion for model selection related to the better known Akaike Information Criterion (AIC) and Bayesian (or Schwarz) Information Criterion (BIC). Here we use type V DIC, which we investigate as a model selection criterion for sequence segmentation in [31]. DICV is defined:
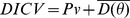
where 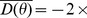 average of log-likelihood over the set of segmentations sampled by MCMC and 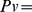 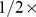 variance of log-likelihood over the set of samples.

Models with smaller DICV are preferred; however, it often happens that there is no clear minimum. In general we select the value of 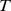 which corresponds to the first local minimum of DICV. However, a subjective judgement is used when it appears obvious that the DICV values continue to decrease significantly with larger values of 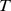. For a detailed discussion of using information criterion to select the number of classes, see [31].

#### Procedure 2: Investigating the stability of classes

In this procedure the model selected was the model with the largest number of classes in which each class was considered stable. Stability of classes was assessed based on time-series plots of conservation levels and GC content versus sample number. Classes which were highly variable in either GC content or conservation level were deemed unstable (again this involved a subjective judgement). As previously mentioned, the mixture proportions of the classes was used as a second criteria to assess the selected model, and a model with a smaller number of classes was selected if any of the classes were deemed empty.

### Class Profiling

The positions of segments in each of the segment classes in each of models chosen by Procedure 2 were recorded in BED format (BED files submitted with supplementary material), with genomic coordinates relative to the *D. melanogaster* genome (release R5.33). The *D. melanogaster* sequence corresponding to each segment for each of the segment classes was also extracted in fasta format. We defined segments as belonging to a particular class as contiguous runs of at least eight sequence positions at which the posterior probability of belonging to the given class is 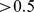. The use of the threshold (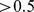) is discussed in [13], and is demonstrated to be an effective compromise between false negative and false positive allocation of positions to segment classes.

#### MEME motif identification

MEME [43] was used to search for motifs shared by segments from the profiled classes. We allowed the option of zero or one motif per sequence in all queries, with a maximum motif size of 20 base pairs and for the reverse complement of each sequence to be considered. For each motif identified by MEME with an E-value 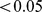, TOMTOM [44] (web interface: http://meme.nbcr.net/meme/cgi-bin/tomtom.cgi) was then used to search for similar motifs in each of four motif databases: “All Drosophila”; “JASPAR-insects”; “All Vertebrates”; “RNA-binding motifs” (descriptions of the motif databases are found at the web interface). Motifs reported by TOMTOM with an E-value 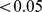 were considered significantly similar.

#### Annotation enrichment

Drosophila 3′ UTR annotations were obtained from UTRdb [16] and PicTar output [32], then segment classes were tested for enrichment in each of the annotation types. The Drosophila subset of the UTRdb dataset of annotations (UTRef) was obtained from http://ebi.edu.au/ftp/databases/UTR/data/. All Drosophila annotation in UTRef are based on pattern similarity identified using the tool UTRscan. PicTar is a program for predicting miRNA binding sites from multiple species alignments, sites predicted in Drosophila were obtained from http://dorina.mdc-berlin.de/rbp_browser/dm3.html.

The positions of annotations in *D. melanogaster* were compared with the positions of each of the segment classes of the 15-class model of the *D. melanogaster* versus *D. simulans* 3′ UTR alignment. For each annotation type we test whether there is evidence for enrichment of that annotation type in our segment classes. For the null hypothesis of no enrichment, the expected number of annotations in each segment class is based on the proportion of the *D. melanogaster* sequence covered by each segment class. The bagFFT algorithm [45] (web interface: http://www.cs.cornell.edu/w8/~niranjan/llr.html) was used to calculate p-values for an exact multinomial goodness-of-fit test. Annotation types with p-value 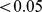, after Bonferroni correction for multiple testing, are considered significant. Only annotation types with more than one match in the segment classes are considered for testing. For annotation types with significant p-values, classes containing more occurrences of that type than expected are considered enriched in that element.

## Supporting Information

Figure S1 **DICV values for segmentation of 3-way alignment.** DICV values obtained using 1–20 segment classes for *D. melanogaster*, *D. simulans* and *D. yakuba* 3′ UTR alignment. The 14-class model was selected as minimum DICV has occurred at class 14.
(TIFF)

Figure S2 **GC content versus conservation level for models selected for 3-way alignment.** GC content of *D. melanogaster* versus the proportion of alignment matches, for each model selected for the 3-way 3′ UTR alignment. A) 14-class model selected by Procedure 1 and B) 15-class model selected by Procedure 2. The different colours represent different classes, and each class is plotted for the post burn-in samples. This plot was used to access the convergence of the selected models.
(TIF)

Figure S3 **DICV values for the control sequence.** DICV values were obtained for an artificially generated sequence having only one class of segments. The minimum DICV has occurred at 1-class; therefore justifies models selected by Procedure 1.
(TIFF)

Figure S4 **Conservation level vs sample number for control sequences.** Figure shows time-series plots of conservation level versus sample number for segmentations of artificially generated control sequence with A) 1 segment class and B) 2 segment classes.
(TIF)

Table S1 **Model comparisons - Procedure 1 versus Procedure 2.** Comparing characteristics of the two models selected by Procedure 1 and Procedure 2 (12-class model and 15-class model respectively) for 3′ UTR alignment of *D. melanogaster* versus *D. simulans*.
(XLSX)

Table S2 **Types of motif identified in 12-class model of D. melanogaster vs D. simulans alignment.** Types of motif identified in *D. melanogaster* versus *D. simulans* 12-class model selected by Procedure 1.
(XLSX)

Table S3 **Types of motif identified in 15-class model of D. melanogaster versus D. simulans alignment.** Types of motif identified in *D. melanogaster* versus *D. simulans* 15-class model selected by Procedure 2.
(XLSX)

Table S4 **Types of motif identified in 16-class model of D. melanogaster versus D. yakuba alignment.** Types of motif identified in *D. melanogaster* versus *D. yakuba* 16-class model selected by Procedure 2.
(XLSX)

Table S5 **Class comparisons of** 3′ **UTR pairwise alignments.** Comparison of change-point character frequencies in each of the classes identified by Procedure 2 for each pairwise alignment of *D. melanogaster* (D. mel), *D. simulans* (D. sim), and *D. yakuba* (D. yak) 3′ UTRs. Classes from different models with similar character frequencies are grouped together.
(XLSX)

Table S6 **Class comparisons of** 3′ **UTR pairwise and 3-way alignments.**
(XLSX)

Table S7 **Enrichment of PicTar miRNA targets in segment classes.**
(XLSX)

Table S8 **Enrichment of UTRdb motifs in segment classes.**
(XLSX)

File S1 **Positions of segments for the 15-class model of D. melanogaster versus D. simulans alignment.**
(BED)

File S2 **Positions of segments for the 16-class model of D. melanogaster versus D. yakuba alignment.**
(BED)

File S3 **Positions of segments for the 15-class model of 3-way D. melanogaster, D. simulans, D. yakuba alignment.**
(BED)
