# Supplementary figures and images for: Drosophila 3′ UTRs Are More Complex than Protein-Coding Sequences
Source: PLoS One. 2014 May 13;9(5):e97336. doi: 10.1371/journal.pone.0097336 (PMC4019593; doi:10.1371/journal.pone.0097336)

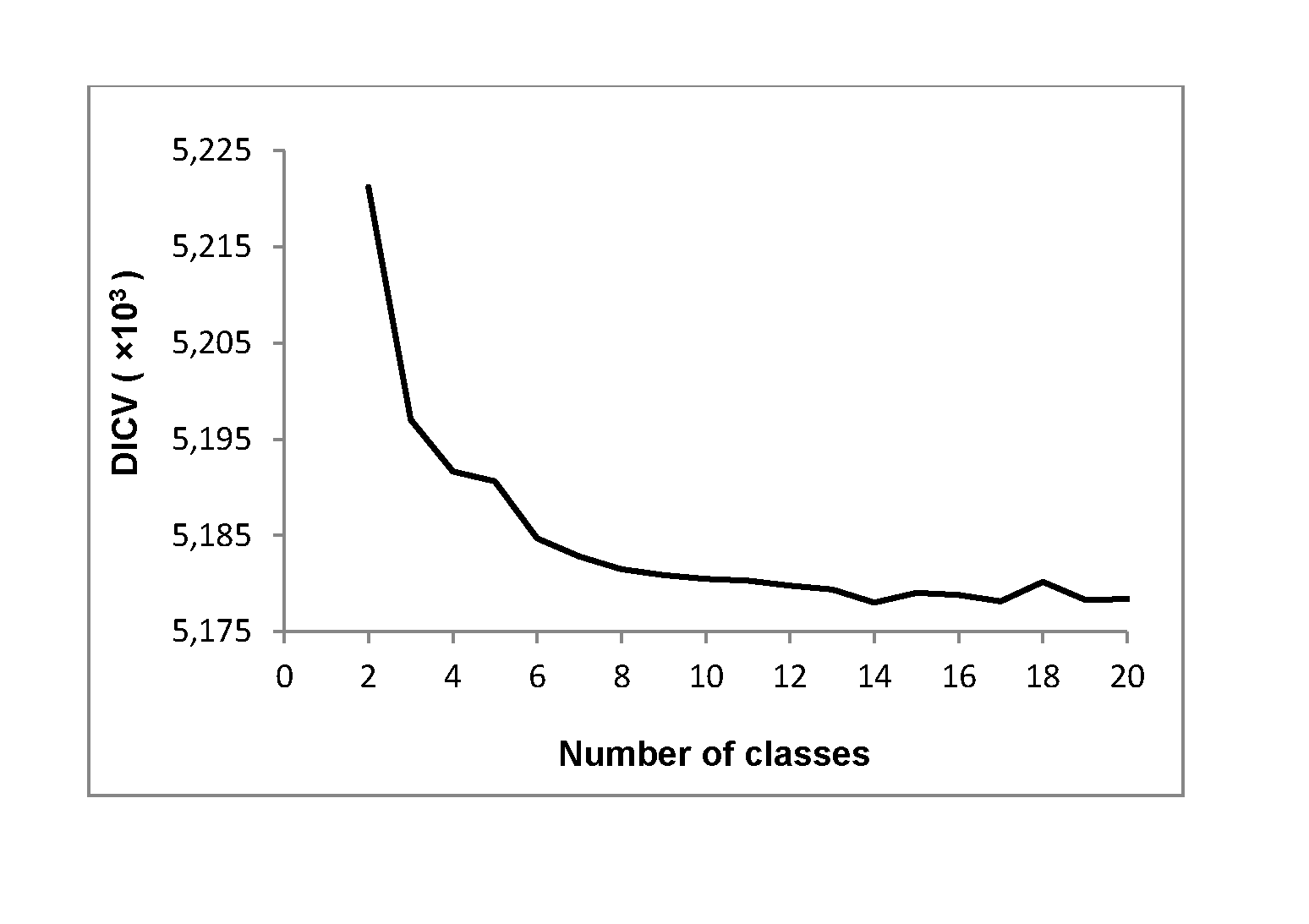

Supplement: Figure S1 — DICV values for segmentation of 3-way alignment. DICV values obtained using 1–20 segment classes for D. melanogaster, D. simulans and D. yakuba 3′ UTR alignment. The 14-class model was selected as minimum DICV has occurred at class 14. (TIFF) [file pone.0097336.s001.tiff]

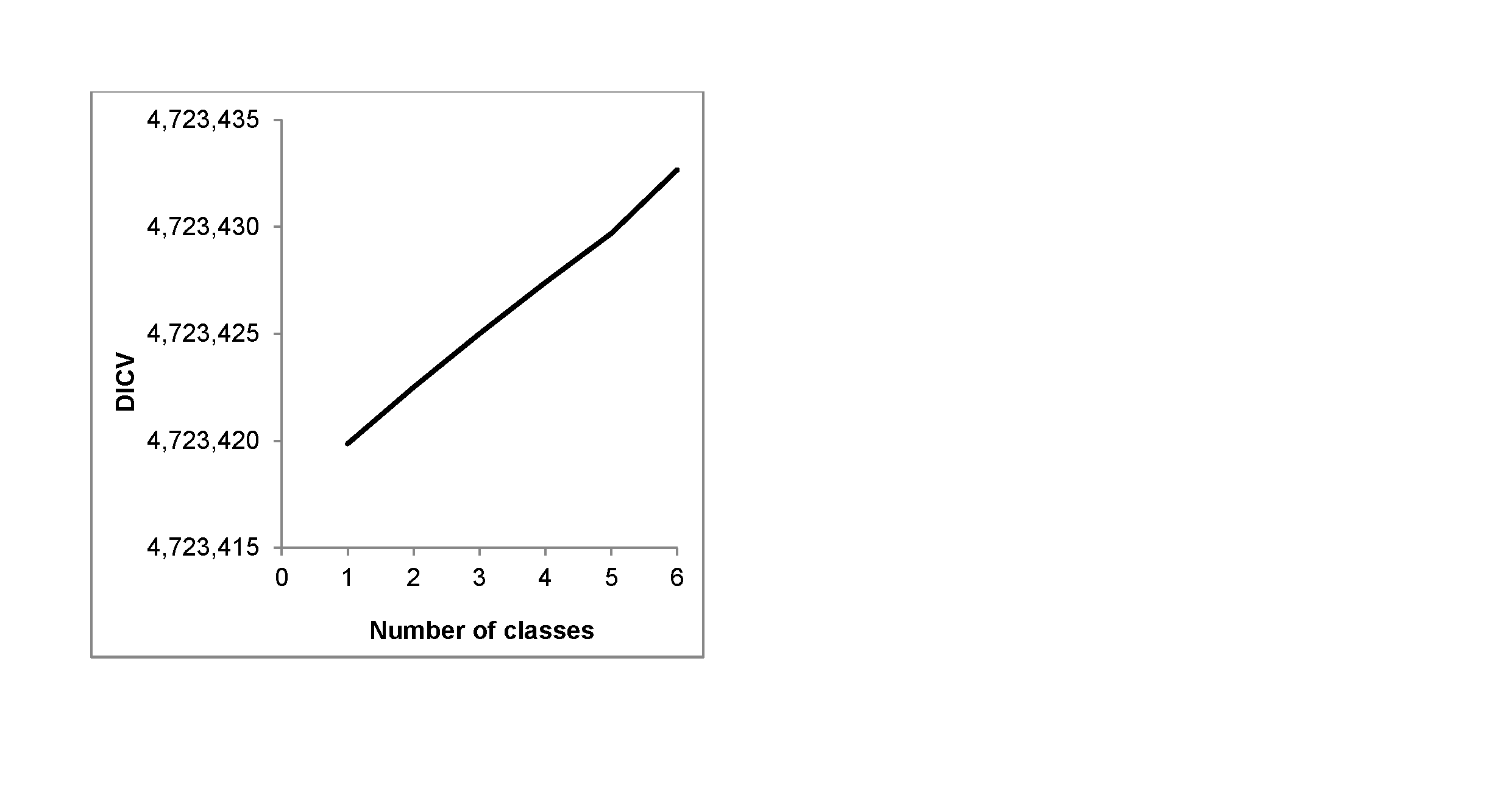

Supplement: Figure S3 — DICV values for the control sequence. DICV values were obtained for an artificially generated sequence having only one class of segments. The minimum DICV has occurred at 1-class; therefore justifies models selected by Procedure 1. (TIFF) [file pone.0097336.s003.tiff]

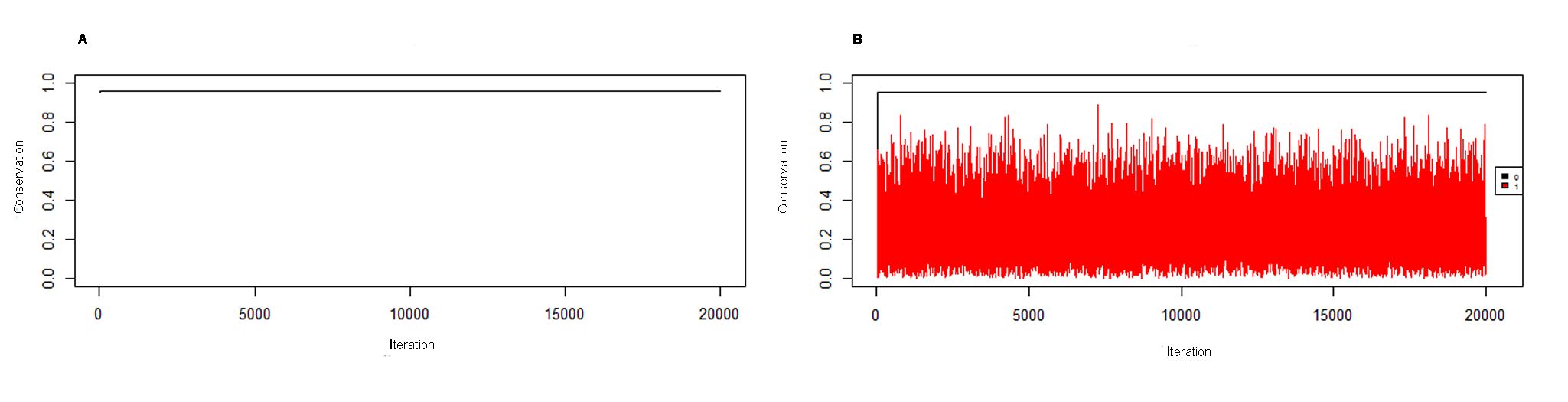

Supplement: Figure S4 — Conservation level vs sample number for control sequences. Figure shows time-series plots of conservation level versus sample number for segmentations of artificially generated control sequence with A) 1 segment class and B) 2 segment classes. (TIF) [file pone.0097336.s004.tif]
